# Are the factors associated with overweight/general obesity and abdominal obesity different depending on menopausal status?

**DOI:** 10.1371/journal.pone.0245150

**Published:** 2021-02-04

**Authors:** Jyu-Lin Chen, Jia Guo, Ping Mao, Jundi Yang, Shan Jiang, Wei He, Chen-Xi Lin, Kathy Lien

**Affiliations:** 1 Department of Family Health Care Nursing, University of California, San Francisco, California, United States of America; 2 Xiangya School of Nursing, Central South University, Changsha, China; 3 Nursing Department, Third Xiangya Hospital, Central South University, Changsha, Hunan, China; 4 Nursing Department, Zhongda Hospital, Southeast University, Jiangsu, Nanjing, China; Universidad Nacional Autonoma de Mexico, MEXICO

## Abstract

Rapid modernization in China has impacted the daily lives and health of women, including a rise in obesity. However, little is known about the impact of menopausal status, behavior, and psychosocial factors on the risk of obesity for rural women in China. The aim of this study is to identify risk factors, including demographic information (education, family history of T2DM, menopausal status), obesity-related behavior, and psychosocial factors associated with overweight/general obesity and abdominal obesity. In a cross-sectional study design, participants had their weight, height, and waist circumference measured and completed questionnaires regarding family demographics, obesity-related health behaviors (physical activity, diet, sleep), and psychosocial information (stress, social support, and self-efficacy related to physical activity and healthy diet). A total of 646 women were included in this study; 46.6% were overweight/generally obese, and 48% had abdominal obesity. Postmenopausal women had a higher prevalence of general and central obesity. Regular physical activity decreased the risk for overweight/general obesity and abdominal obesity (OR = .41 and .31, respectively, p = .04) in premenopausal women. Postmenopausal women who had not breastfed their infants and reported moderate/high-stress had a higher risk for overweight/general obesity (OR = 3.93, and 2, respectively) and those who reported less than 6 hours of sleep per day increased their risk for abdominal obesity (OR = 2.08). Different factors associated with obesity were found in Chinese women, depending on menopausal status. Future studies should examine the impact of menopause on a woman’s risk for obesity, as well as develop tailored interventions to improve health, well-being and reduce the risk of obesity.

## Introduction

Obesity is a global health concern that has reached pandemic proportions. It is estimated that by 2030, 38% of the global adult population will be overweight, and 20% will be obese, based on body mass index (BMI) [1]. In China, the prevalence of overweight and obesity among adults has increased from 20% in 1992 to 42% in 2012, according to the Chinese BMI standard [2]. A similar increase in the prevalence of abdominal obesity measured by waist circumference (WC; waist circumference > 90cm for males and 80cm for females) was found in China. The prevalence of abdominal obesity among 174,640 Chinese adults in China increased from 27.5% in 2007 to 31.5% in 2014 [3]. Recent data from the China Health and Nutrition Survey (CHNA) with 12,543 adults found that the age-adjusted prevalence of overweight, general obesity, and abdominal obesity were 38.8%, 13.99%, and 43.15%, respectively, with a significantly higher prevalence of abdominal obesity among women compared to men across all age groups [4]. These trends are very concerning, especially among Chinese women, as obesity is associated with numerous comorbidities, including hypertension, cardiovascular disease, dyslipidemia, type 2 diabetes mellitus (T2DM), and certain cancers, including breast and endometrial cancers in women [5,6].

Factors that increase the individual’s risk for obesity include an unhealthy diet, physical inactivity, poor sleep, and a high level of stress [2,7–9]. In China, the association between unhealthy diet and obesity may be due to increased urbanization, which has led to the more widespread consumption of a Western diet that is high in energy, high in fat, low in fiber [2], and consists of more protein products such as red meat, poultry, fish, eggs, and dairy [8,9]. Low levels of physical activity is another risk factor for obesity. In a 20-year longitudinal study, high levels of physical activity were associated with smaller gains in BMI and waist circumference, compared to lower levels of activity [10]. Yet, a recent China Health and Retirement Longitudinal Study found that only about 35% of adults in China reported physical activity at least 10 minutes continuously per day, and a low level of physical activity significantly increased the risk for obesity [11]. While the number of adults in China participating in minimum leisure-time physical activity recommendations, including sports, exercising, or recreational walking, has increased over the past two decades (17.2% in 2000, 18.1% in 2005, and 22.8% in 2014) [3], fitness indicators for lung capacity, muscular strength, flexibility, and balance remain low for all age groups [3]. This may be because increased urbanization in China has led to a decline in other indicators of physical activity, such as occupational and domestic activity, which are important contributors towards overall energy expenditure [12,13].

Other factors that may influence weight gain include sleep and stress. Several studies have found that less sleeping time and poor sleep quality increase the risk of obesity in Chinese adults [14–16]. In a meta-analysis of longitudinal studies, short sleep duration was shown to affect appetite-regulating hormones, leptin, and ghrelin [17], which may explain the association between short sleep duration and obesity [18]. Further, exposure to stress increases the risk of obesity because it can lead to physiological changes in the hypothalamic-pituitary-adrenal axis and secretion of cortisol, promoting processes that lead to weight gain [19–21]. On the other hand, better social support for physical activity and a healthy diet has been found to reduce the risk of obesity, including in Chinese women [22,23]. Moreover, positive self-efficacy about one’s ability to be active and consume a healthy diet is also associated with healthy lifestyle improvements and a reduced risk for obesity, including in Chinese adults [24,25].

There are sex-specific differences in how men and women gain weight and are affected by weight gain. Fat storage in women tends to occur subcutaneously, whereas fat storage in men tends to occur viscerally [26–28]. Although the menopausal transition is not entirely responsible for weight gain in mid-life, changes in the hormonal milieu at menopause have been shown to more rapidly increase fat mass and redistribute fat to the abdomen which increases the risk for abdominal obesity [29]. In addition to menopause, breastfeeding has been suggested to be associated with obesity in women. A systematic review and meta-analysis found that breastfeeding was inversely associated with overweight and obesity with a pooled odds ratio of 0.74 [30]. However, there is limited evidence on the impact of breastfeeding, behavioral factors (diet, physical activity, sleep) and psychosocial factors (stress, social support, and self-efficacy) on obesity (both general and abdominal) based on menopausal status in rural Chinese women.

The purposes of this study were to investigate the following questions among rural Chinese women: 1) the prevalence of overweight/general obesity and abdominal obesity; 2) obesity-related behaviors (physical activity, fruit/vegetable intake, and sleep), and psychosocial factors (stress, social support, and self-efficacy); and 3) risk factors, including demographic information (education, family history of T2DM, menopausal status), obesity-related behavior, and psychosocial factors associated with overweight/general obesity and abdominal obesity.

## Materials and methods

The Institutional Review Board for human research from a local university approved this study (No.2018028). This study utilized a cross-sectional study design. Study participants from rural areas in Hunan province (located in the central south of mainland China) were recruited. The inclusion criteria for study participants included: 1) of the biological female sex, 2) 18 years and older, 3) able to speak Mandarin, and 4) living in a rural area in Hunan province. Individuals who are currently pregnant, could not perform their regular daily activities or on a restricted diet and limited physical activity due to illness (such as dialysis, under chemotherapy, stroke) were excluded from this study. Data were collected between July and October 2018.

Study participants were recruited from three county-level general hospitals and two central township hospitals in Changsa, Hunan province in China, at the free cancer screening programs (breast cancer and cervical cancer) for female residents. Each potential eligible participants were approached by the trained research assistants who explained the purpose of the study. Interested participants were given a content form to complete. After obtaining written informed consent, trained research assistants at the study sites collected height, weight, and waist circumference. Study participants who consented to participate in this study also completed several surveys regarding their basic demographic information, obesity-related health behaviors, self-efficacy regarding diet and physical activity, and social support related to diet and physical activity. The research took place in the screening programs at the three county-level general hospitals and two central township hospitals in Hunan Province. Study participants are considered representative of the female population in the rural Hunan province in China.

### Measurements

#### Demographics

The demographic survey included sociodemographic information such as age, education level, and annual family income, immediate family history of type 2 diabetes (T2DM), and menstrual histories, such as menarche age, menstrual cycle, and menopause status.

#### Body mass index (BMI)

Study participants had their weight and height measured with shoes off and light clothes on. BMI was calculated as follows: BMI = weight (kg)/height (m)^2^. The overweight and obesity definitions were based on the China criteria: BMI <18.5kg/m^2^ is considered as underweight, 18.5kg/m^2^ ≤ BMI <24.0kg/m^2^ is considered normal weight, 24.0kg/m^2^ ≤ BMI <28.0kg/m^2^ is considered overweight, BMI ≥28.0kg/m^2^ is considered obese [4]. The height and weight instruments were calibrated before each measurement. Height was accurate to 0.1cm and weight was accurate to 0.1kg.

#### Waist circumference

Measured at the horizontal circumference of the lower edge of the costal arch and the midpoint of the condylar line using a tape measure with an accuracy of 0.5 cm. Central obesity is defined as a waist circumference ≥ of 80.0 cm for Chinese women [4].

#### Obesity-related behaviors

Items regarding obesity-related behaviors such as vegetable and fruit intake (>5 servings/per day), regular physical activity (a total of 150 minutes per week), sleep (>6 hrs/per day), and breastfeeding history were adopted from the Canadian Diabetes Risk Questionnaire for the Chinese population survey (CHINARISK). The CHINARISK survey has been used in China with adequate reliability (test-retested reliability = .98) and validity (sensitivity = .73 and specificity = .63) [31].

#### Self-efficacy for diet and physical activity

The 8-item survey used in the study was adapted from the Eating Habits and Exercise Confidence Survey developed by Sallis and colleagues (Sallis et al.,1988). Participants were asked about their level of confidence in engaging in activities related to a healthy diet and regular physical activities. The survey uses 5 points for scoring (0- not confident to 5- very confident). A higher score indicates a higher level of self-efficacy. The Cronbach’s alpha range for the exercise self-efficacy scale is 0.87 to 0.97, and the Cronbach’s alpha range for the diet self-efficacy scale is 0.93 to 0.95 [32].

#### Social support for diet and physical activity

The scale used in the study was adapted from Sallis et al., Social Support for Diet and Exercise Survey [33]. The scale contains six items, of which three items are to evaluate the support of diet and exercise provided by family members. The other three items are to evaluate the support of diet and exercise provided by friends. The dietary, social support options are rated on a 4-point scale: 0 (never), 1 (sometimes), 2 (frequently), or 3 (always). The sports social support option is rated on a 5-point scale: 0 (never), 1 (rarely), 2 (occasionally), 3 (frequently), and 4 (always). Each item is added separately to get a total score. Higher scores indicate more social support for the participant. The dietary, social support scale Cronbach’s alpha ranges from 0.80 to 87, and the social support scale Cronbach’s alpha ranges from 0.84 to 0.91 [33].

#### Perceived stress scale (PSS)

This scale is used to measure the degree of perceived stress that the subject perceives in the past month. It is divided into three versions of 14 items (PSS-14), ten items (PSS-10), and four items (PSS-4). The 14-item version was used in this study. The study participants are asked to respond to questions on how often they have felt (such as upset, nervous, anger, difficulties) over the past one month on a five-point Likert scale (0 = never to 4 = very often), indicating. The higher the score, the higher the perceived pressure. Good psychometric properties have been reported in the Chinese population [34].

### Data analysis

Descriptive analyses were used to describe sociodemographic characteristics and obesity-related health behaviors. Chi-square tests were performed to examine differences in the prevalence of obesity and obesity-related health behaviors based on menopause status (premenopausal or postmenopausal). Four multivariate logistic regression analyses were performed to determine the odds ratios (ORs) and 95% confidence intervals (CIs) between the above variables and overweight/general obesity and abdominal obesity based on menopause status (premenopausal or postmenopausal). Each analysis was fit first with demographic variables (education and family history of diabetes) as categorical variables in model 1. In model 2, additional obesity-related health behaviors (breastfeeding, regular physical activity, having five servings of fruit and vegetable intake per day, sleep) as categorical variables were added to the model. In model 3, demographic variables, obesity-related health behaviors, and additional psychosocial variables (stress, self-efficacy related to diet and physical activity, and social support for diet and physical activity) were included in the final model. All statistical analyses were performed with SPSS 26.0 for Windows with a significance value set at p < .05.

## Results and discussion

### Demographic

A total of 646 women with a mean age of 50.7 years (SD = 3.1) were included in this study. Out of this group, 344 women (53.3%) self-reported at the postmenopause stage (mean age = 55.08, SD = 6.21) and 302 women (46.7%) at the premenopause stage (mean age = 45.7, SD = 5.0). The majority of the participants were married (97.3%), had children (99.7%), received less than high school education (85.3%), were employed (89.2%), and about half of the study participants reported a family monthly income of less than 3,000 yuan, about USD 458 (51.2%). There was a significantly higher portion of premenopausal women who received high school and above high school education compared to postmenopausal women (*X*^2^ = 7.35, p = .007, Table 1).

**Table 1:** Descriptive data.

Variables	All women N/mean (% or SD)	Pre-Menopause N/mean (% or SD)	Post Menopause N/mean (% or SD)	X^2^ or t (P)
BMI (>24)	301 (46.6%)	125 (41.5%)	176 (51.2%)	7.02 (.008)
WC (>80cm)	310 (48%)	120 (43%)	180 (52.3%)	5.47 (.019)
Education (> junior high)	95 (14.7%)	56 (19%)	39 (11.3%)	7.35 (.007)
Family hx of T2DM (yes)	526 (81.4%)	239(89.5%)	282(89.5%)	.0 1 (.997)
Breastfeeding (Yes)	615 (95.2%)	291 (96.4%)	324 (94.2%)	1.53 (.215)
Regular physical activity (yes)	280 (43.3%)	120(41%)	155(45.5%)	1.29 (.254)
F/V intake (>5 servings/day)	307(47.5%)	146(49.8%)	156(45.6%)	1.12 (.289)
Sleep (< 6hr/day)	168 (26%)	47(16%)	118(34.4%)	27.97 (<.001)
Life stress (moderate/high)	143 (22.1%)	90 (29.8%)	60 (17.4%)	9.55 (.002)
Dietary self-efficacy (mean)	9.64 (3.78)	9.71 (3.93)	9.55 (3.665)	.53 (.593)
Physical activity self-efficacy (mean)	7.24(4.6)	7.17(4.38)	7.36(4.746)	-.50 (0.165)
Social support for diet (mean)	2.95 (.36)	2.93 (.35)	2.97 (.37)	-1.29 (.20)
Social support for physical activity (mean)	1.61 (1.00)	1.66 (.95)	1.57 (1.04)	1.21 (.26)

### Prevalence of overweight/general obesity and abdominal obesity

The mean BMI was 23.85 (SD = 3.09), and the mean waist circumference was 79.35cm (SD = 8.72). There were 301 women with BMI greater than 24 kg/m^2^ (46.6%) and 310 women (48%) with a waist circumference greater than 80cm (see Table 1). Postmenopausal women had a statistically higher prevalence of overweight/general obesity (51.2% vs. 41.5%) and abdominal obesity than premenopausal women (52.3% vs. 43%) (Table 1).

### Obesity-related behaviors

A majority of the women had breastfed their infants (95.2%), 43.3% reported regular physical activity, 47.5% ate more than five servings of fruits and vegetables per day, and 26% reported sleeping less than 6 hours per day. About 22.1% of women reported moderate to high levels of stress. Postmenopausal women reported less sleep (34.4% vs. 16%) and lower levels of moderate/high stress (17.4% vs. 29.8%) compared to premenopausal women (Table 1). There was no difference between premenopausal and postmenopausal women in physical activity, fruit and vegetable intake, self-efficacy related to diet and physical activity, and social support (Table 1).

### Risk factors for overweight/general obesity

For premenopausal women, models 1 and 2 did not identify demographic and obesity-related behaviors factors related to overweight/general obesity. In model 3, women who reported regular physical activity reduced their risk for overweight/general obesity compared to women who did not report regular physical activity (OR = .41, 95% CI = .17-.97) (Table 2).

**Table 2:** Logistic regression analysis of determinants of BMI >= 24 for premenopausal women.

Variables	Model 1 (sociodemographic characteristics)				Model 2 (sociodemographic characteristics+behavior)				Model 3 (sociodemographic characteristics+ behavior +psychosocial)			
	B	OR	95%CI	*p-v*alue	B	OR	95%CI	*p-*value	B	OR	95%CI	*p-*value
Education (0> junior high)	.30	1.35	.65, 2.82	.42	.53	1.71	.82, 3.5	.15	.55	1.73	.81, 3.67	.16
Family hx of T2DM (0 = no)	-.51	.60	.27, 1.34	.21	.71	2.0	.82, 5.0	.13	.86	2.35	.91, 6.1	.08
Breastfeeding (0 = yes)					1.73	5.66	.60, 49.9	.12	1.70	5.5	.61, 49.58	.13
**Regular physical activity (0 = no)**					**-.71**	**.49**	**.23, 1.06**	**.07**	**-.89**	**.41**	**.17, .97**	**.04**
F/V intake (0<5 servings/day)					-.32	.72	.42, 1.25	.25	-.43	.65	.37, 1.15	.14
Sleep (0< 6hr/day)					-.58	.56	.28, 1.1	.09	-.60	.55	.27, 1.10	.09
Moderate/high stress (0 = no)									-.05	.95	.50, 1.79	.87
Dietary self-efficacy									.78	1.08	.99, 1.17	.06
Physical activity self-efficacy									.04	1.04	.96, 1.13	.32
Social support for diet									-.50	.61	.26, 1.39	.24
Social support for physical activity									-.13	.87	.62, 1.24	.45

For postmenopausal women, no demographic factor was found to be related to overweight/general obesity. In model 2, women who did not breastfeed their infants had an increased likelihood of being overweight/generally obese (OR = 3.93, 95% CI = .1.25–12.4). In model 3, women who did not breastfeed their infants (OR = 4.54, 95% CI = 1.37–15) and reported moderate/high stress were more likely to be overweight and obese (Table 3).

**Table 3:** Logistic regression analysis of determinants of BMI >= 24 for postmenopausal women.

Variables	Model 1 (sociodemographic characteristics)				Model 2 (sociodemographic characteristics+Behavior)				Model 3 (sociodemographic characteristics+ behavior +psychosocial)			
	B	OR	95%CI	*p-v*alue	B	OR	95%CI	*p-*value	B	OR	95%CI	*p-*value
Education (0> junior high)	.30	.74	.36, 1.54	.42	.19	.82	.39, 1.74	.61	.33	.72	.33, 1.58	.41
Family hx of T2DM (0 = no)	-.51	1.66	.76, 3.66	.21	.39	1.48	.67, 3.3	.34	.42	1.52	.67, 3.45	.32
**Breastfeeding** (0 = yes)					**1.37**	**3.93**	**1.25, 12.40**	**.02**	**1.51**	**4.54**	**1.37, 15.0**	**.01**
Regular physical activity (0 = no)					.21	1.24	.72, 2.12	.44	.27	1.31	.70, 2.47	.40
F/V intake (0<5 servings/day)					.08	1.09	.66, 1.77	.74	.21	1.23	.73, 2.09	.44
Sleep (0< 6hr/day)					.41	1.50	.91, 2.47	.11	.46	.64	.38, 1.07	.09
**Moderate/high stress (0 = no)**									**-.69**	**.50**	**.26 .97**	**.04**
Dietary self-efficacy									.02	1.02	.95, 1.09	.69
Physical activity self-efficacy									.02	1.02	.95, 1.09	.53
Social support for diet									-.73	1.08	1.05, 3.11	.09
Social support for physical activity									-.26	.77	.57, 1.04	.09

### Risk factors for abdominal obesity

In premenopausal women, no demographic variables were identified in model 1. Women who reported regular physical activity and consuming five servings of fruits and vegetables decreased their risk for abdominal obesity (OR = .31, 95% CI = .13-.75 and OR = .57, 95% CI = .32-.99 respectively) (Table 4).

**Table 4:** Logistic regression analysis of determinants of WC >= 80cm for premenopausal women.

Variables	Model 1 (sociodemographic characteristics)				Model 2 (sociodemographic characteristics+Behavior)				Model 3 (sociodemographic characteristics+ behavior +psychosocial)			
	B	OR	95%CI	*p-v*alue	B	OR	95%CI	*p-*value	B	OR	95%CI	*p-*value
Education (0< junior high)	.14	1.15	.59, 2.23	.69	.08	1.09	.54, 2.17	.81	.16	1.17	.57, 2.40	.67
Family hx of T2DM (0 = no)	.12	1.13	.48, 2.64	.79	.25	1.28	532, 3.11	.59	.17	1.19	.47, 2.99	.72
Breastfeeding (0 = yes)					.24	1.27	.26, 6.11	.77	.25	1.28	.26, 6.34	.76
**Regular physical activity (0 = No)**					**-1.05**	**.35**	**.16, .79**	**.01**	**-1.18**	**.31**	**.13, .75**	**.01**
**F/V intake (0<5 servings/day)**					-.50	.61	.35, 1.04	.07	**-.57**	**.57**	**.32, .99**	**.04**
Sleep (0< 6hr/day)					-.12	.89	.45, 1.75	.73	-.11	.90	.46, 1.80	.76
Moderate/high stress (0 = no)									.43	1.54	.83, 2.85	.17
Dietary self-efficacy									.05	1.06	.97, 1.14	.19
Physical activity self-efficacy									-.01	.99	.92, 1.08	.86
Social support for diet									-.38	.68	.30, 1.56	.37
Social support for physical activity									.15	1.16	.82, 1.64	.42

In postmenopausal women, those who slept less than 6 hours per day were more likely to have abdominal obesity (OR = .2.08, 95% CI = .1.26–3.42 in model 2) OR = 2.17, 95% CI = 1.30–3.6 in model 3) compared to women who slept less than 6 hours per day. No other demographic variables, behavior, and psychosocial variables entered the model (Table 5).

**Table 5:** Logistic regression analysis of determinants of WC >= 80cm for postmenopausal women.

Variables	Model 1 (sociodemographic characteristics)				Model 2 (sociodemographic characteristics+Behavior)				Model 3 (sociodemographic characteristics+ behavior +psychosocial)			
	B	OR	95%CI	*p-v*alue	B	OR	95%CI	*p-*value	B	OR	95%CI	*p-*value
Education (0< junior high)	-.45	.64	.30, 1.35	.24	-.42	.66	.30, 1.42	.29	-.51	.60	.27, 1.34	.21
Family hx of T2DM (0 = no)	.37	1.45	.66, 3.09	.36	.22	1.25	.56, 2.80	.59	.20	1.23	.54, 2.78	.62
Breastfeeding (0 = yes)					.65	1.91	.71, 5.18	.20	.69	1.98	.71, 5.66	.19
Regular physical activity (0 = No)					-.14	.87	.51, 1.50	.62	.14	1.15	.61, 2.15	.67
F/V intake (0<5 servings/day)					.01	1.0	.62, 1.65	.97	.14	1.15	.68, 1.93	.61
**Sleep (0< 6hr/day)**					**.73**	**2.08**	**1.26, 3.42**	**.004**	**.77**	**2.17**	**1.30, 3.6**	**.003**
Moderate/high stress (0 = no)									.13	1.14	.59, 2.2	.70
Dietary self-efficacy									-.01	.99	.92, 1.06	.73
Physical activity self-efficacy									-03	.97	.91, 1.03	.32
Negative social support for diet									.26	1.3	.67, 2.54	.44
Social support for physical activity									-.08	.92	.69, 1.24	.59

## Discussion

The purpose of this study was to identify factors associated with overweight/general and abdominal obesity in rural women in China. We found a high prevalence of overweight/ general/obesity (46.6%) and abdominal obesity (48%) in our participants, with a significantly higher prevalence in postmenopausal women compared to premenopausal women. This study also found that less than 50% of women reported having more than five servings of fruits/vegetables and meeting the regular physical activity recommendations, while about a quarter of women reported sleep less than 6 hours per day and reported moderate and/or higher level of stress. Among premenopausal women, having regular physical activity decreased obesity risk compared to those who reported no regular physical activity. For postmenopausal women, women who did not breastfeed their infants and reported moderate/high level of stress increased their risk for overweight/general obesity, and those who slept less than 6 hours/per day increased their risk for abdominal obesity.

The high prevalence of overweight/general obesity and abdominal obesity in our study is alarming, especially among postmenopausal women a Our study found that about 50% of postmenopausal women had BMI greater than 24 kg/m2 and waist circumference greater than 80cm. The prevalence of abdominal obesity is similar to the 2011 China Health and Nutrition Survey (CHNS) data, suggesting that the age-adjusted prevalence of abdominal obesity was 51.7% among Chinese women [35]. Another study also found a significant increase, with the age-adjusted prevalence of overweight and general obesity from 30.22% to 49.22%, and abdominal obesity from 29.75% to 50.75% in Chinese women from 1989 to 2011 [4]. As obesity increases the risk for many other health conditions, including T2DM, cardiovascular diseases, and certain types of cancer [5,36], intervention that aims to reduce obesity is critical to women’s health, especially postmenopausal women.

Our study found that less than 50% of the women reported eating more than five servings of fruits/vegetables and meeting the level of physical activity recommendations. The low levels of physical activity in Chinese women and their not meeting dietary recommendations have been reported in several studies [13,37–39]. Chinese women reported a decline in physical activity levels across all age groups (25 to 48 years old) from 1991 to 2011 [40]. The decline in physical activity and not meeting recommended fruit and vegetable intake may be due to China’s modernization, which has impacted lifestyles with increased sedentary time, screen time, and access to a Western diet that also increases the risk for obesity.

Our study found that among premenopausal women, those who do not have regular physical activity and do not consume at least five servings of fruits and vegetables per day are almost twice as likely to be overweight/general obesity and have abdominal obesity compared to those who met these recommendations. There is evidence to support that low levels of physical activity and an unhealthy diet increases the risk of obesity in women [7,9,41]. Studies have suggested that premenopausal women with abdominal obesity have an increased risk of breast cancer by 1.09 fold and endometrial cancer by 1.21–1.27 fold [42–45]. Premenopausal women often have dependent children and have high levels of stress and family responsibilities that prevent regular engagement in a healthy lifestyle and early screening activities [46,47]. Because reducing obesity is very difficult, and because of the significantly increased risk for certain gynecologic cancers in obese women, interventions that aim to promote a healthy lifestyle and reduce obesity in premenopausal women are critical.

Our study found that postmenopausal women who did not breastfeed their infants and reported moderate/high levels of stress were more likely to be overweight/generally obese, and those who slept less than 6 hours were more likely to have abdominal obesity. Further, about 34% of the postmenopausal women reported sleeping less than 6 hours compared to 16% among premenopausal women. Sleep tends to be interrupted in menopause due to hormone fluctuations and declines [48]. Studies have also found that a lack of sleep stress level [17–20,48], and a high level of stress is associated with increased risk for obesity [49]. Interventions that target sleep hygiene and stress management are critical to reducing obesity risk among postmenopausal women in China.

Another interesting finding, which is supported by evidence in the literature, is that breastfeeding has long-term benefits for women’s health, including reducing the risk obesity [30,50]. A meta-analysis of 11 high-quality studies found that breastfeeding decreased the odds of overweight/obesity by 13% [30]. It is not clear why the protective impact of breastfeeding is not seen in the premenopausal women in China. Future studies may need to investigate the role of breastfeeding, along with other risk factors for obesity in Chinese women.

To our knowledge, this is the first study examining factors for both general and abdominal obesity between premenopausal and postmenopausal women in China. Some limitations must be considered. Because of the cross-sectional study design, we cannot determine the causal relationships between risk factors and obesity. This study did not identify the specific stage of menopause using a survey or biological matrix (such as estradiol and FSH level) or examine the impact of contraceptives and menopausal treatment, which may also impact weight gain. A longitudinal design with a clear definition of the menopausal stage and includes treatment that can potentially influence weight should be considered in the future. In addition, obesity-related health behaviors were based on self-reporting, which may increase biases. Future studies may consider other potential risk factors (such as a history of diabetes, hypertension, smoking, and alcohol consumption). Although the study participant may represent women in rural Hunan province, his study also has limited generalizability as participants were recruited from rural areas in Hunan province in China. Despite the listed limitations, our findings are significant for clinical practice and research in reducing health disparities regarding obesity in women in rural China.

## Conclusions

In conclusion, as obesity has become a global epidemic and is linked to many other health issues, understanding risk factors associated with general and abdominal obesity among women in China is a critical step in combating this global health concern. This study found about half of the Chinese women in this study did not meet the recommendations for physical activity and fruit and vegetable intake, and many of them reported having less than 6 hours of sleep and moderate to high level of stress, all important risk factors for obesity. Different factors associated with overweight/general obesity and abdominal obesity were found in Chinese women by menopausal status. Improving healthy lifestyles, especially physical activity, is essential for premenopausal Chinese women, and improved sleep and stress management are crucial for healthy weight control in the postmenopausal group. Health care providers should assess these factors regularly and work with women to develop individualized programs to promote healthy lifestyles and stress management. Future studies should examine the impact of menopause on a woman’s risk for obesity, as well as develop tailored interventions to improve health, well-being and reduce the risk of obesity.

## Supporting information

S1 Data Obesity risk data 2020. (SAV)
